# Associations between Mammary Gland Echotexture and Milk Composition in Cows

**DOI:** 10.3390/ani10112005

**Published:** 2020-10-30

**Authors:** Tomasz Schwarz, Nelia Scheeres, Martyna M. Małopolska, Maciej Murawski, Tristan D. Agustin, Bahareh Ahmadi, Nina Strzałkowska, Patrycja Rajtar, Piotr Micek, Pawel M. Bartlewski

**Affiliations:** 1 Department of Genetics, Animal Breeding and Ethology, Faculty of Animal Science, University of Agriculture in Kraków, 24/28 Mickiewicza Ave., 30-120 Cracow, Poland; rzschwar@cyf-kr.edu.pl; 2 Department of Biomedical Sciences, Ontario Veterinary College, University of Guelph, 50 Stone Rd., Guelph, ON N1G 2W1, Canada; neliascheeres@gmail.com (N.S.); ahmadib@uoguelph.ca (B.A.); pmbart@uoguelph.ca (P.M.B.); 3 Department of Pig Breeding, National Research Institute of Animal Production, 1 Krakowska St., Balice n. 32-083 Cracow, Poland; 4 Department of Nutrition, Animal Biotechnology and Fisheries, Faculty of Animal Science, University of Agriculture in Kraków, 1B Rędzina St., 30-248 Cracow-Krowodrza, Poland; rzmmuraw@cyf-kr.edu.pl (M.M.); patrycja.rajtar@urk.edu.pl (P.R.); rzmicek@cyf-kr.edu.pl (P.M.); 5 College of Veterinary Medicine, University of the Philippines Los Baños, Pedro R. Sandoval Ave., Los Baños, Laguna 4031, Philippines; tbagustin@up.edu.ph; 6 Institute of Genetics and Animal Biotechnology, Polish Academy of Sciences, Jastrzębiec, 36A Postępu St., 05-552 Magdalenka, Poland; n.strzalkowska@ighz.pl

**Keywords:** cow, udder, ultrasonography, image analysis, milk composition

## Abstract

**Simple Summary:**

Transcutaneous ultrasonography of the four quarters of the cow’s mammary gland with the 5-MHz ultrasound transducer combined with computer-assisted analysis of the resultant grey-scale images have the makings of an inexpensive and rapid technique to determine certain physicochemical properties of the pooled milk. The latter include crude protein, casein and lactose content. The relative ease and practically unlimited frequency with which this method can be used in farm settings makes it an attractive alternative to laboratory testing of milk samples. More studies are needed to determine the suitability of this approach for detecting changes in milk chemical composition in animals with mastitis.

**Abstract:**

Thirty clinically healthy Holstein-Friesian cows underwent twice daily machine milking and ultrasonographic examinations of the udder just prior to and after milking. Digital ultrasonographic images of each udder quarter were subjected to computer-assisted echotextural analyses to obtain mean numerical pixel values (NPVs) and pixel heterogeneity (PSD) of the mammary gland parenchyma. The average milk yield and pH were higher (*p* < 0.05) in the morning, whereas crude fat, total solids, solids non-fat and citric acid content were higher (*p* < 0.05) during the evening milking period. Mean NPVs and PSDs of the mammary gland parenchyma were greater (*p* < 0.05) after than before milking. There were significant correlations among echotextural characteristics of the udder and protein percentage, lactose content and freezing point depression determined in the milk samples collected in the morning and crude protein, casein, lactose and solids non-fat in the evening. Our results can be interpreted to suggest that computerized analysis of the mammary gland ultrasonograms has the makings of a technique for estimating non-fat milk constituents in cows. However, future validating studies are necessary before this method can be employed in commercial settings and research. Moreover, significant inter-quarter differences in udder echogenicity may necessitate further echotextural studies of separate quarters.

## 1. Introduction

Milk and assorted milk products from dairy animals are valuable foods that are rich in fat, high quality protein, vitamins, minerals and metabolizable energy [1]. Milking large and small ruminants is of paramount importance in the Global South, where these animals help low-income families overcome poverty and provide a reliable food source to supplement nutrient-poor diets [2]. In developed countries, consumers have started making health-conscious decisions and milk products characterized by desirable fat content are marketable to potential buyers willing to pay a premium price for such food items. Lipids in milk contain a diverse range of fatty acids, including large quantities of saturated and moderate amounts of monounsaturated and polyunsaturated fatty acids, many of which have well-known health benefits [1]. Therefore, dairy farmers responsible for high-quality milk production, most notably organic dairy farmers, and researchers investigating various aspects of lactation, require constant access to technologies that enable frequent, complete and accurate monitoring of milk composition.

Grey-scale ultrasonographic images are composed of numerous brightness elements called pixels corresponding to multiple acoustic interfaces within the examined tissue (the boundaries between regions of different physicochemical properties [3]). Ultrasound transducers contain piezoelectric crystals emitting high-frequency sound waves that are modified as they encounter the acoustic interfaces. Ultrasound beams can be attenuated as they traverse tissues, or they can be scattered or deflected by the scanned objects and their integral components. Scattered or reflected waves received by the transducer determine the relative intensity of each pixel [4]. Numerical pixel values (NPVs), a quantitative measure of pixel brightness, can then be determined objectively using various image analytical software packages. Computerized analysis of ultrasonograms increases precision of measurements and the range of perceivable intensity variations that cannot be detected with the naked eye [5]. The NPV is a unitless parameter that ranges from 0 (absolute black) to 255 (absolute white) and pixel heterogeneity is defined as the standard deviation of NPVs in the area of interest [5]. Both echotextural variables are objective measures of tissue echogenic properties and valuable indicators of corresponding histophysiological changes. In fact, the echotextural attributes of the tissue are the function of its cellular density, hydration, compressibility, macromolecular content and general chemical composition [5,6,7].

It is feasible that milk composition could be assessed or predicted using computer-aided image analyses of the mammary gland. Ultrasound examinations of the udder before milking entail the assessment of various tissue compartments, namely the stromal compartment (made of loose connective tissues containing collagen protein, elastin protein, blood vessels and the milk ductal system) and the parenchymal compartment (containing the secretory epithelium and myoepithelial cells, which are responsible for the synthesis and expulsion of their secretions into milk alveoli [2]). The excretory product contained in the lumen of the alveoli (colostrum or milk) is a third compartment and can be viewed as a non-permanent connective tissue that can easily be “removed” via ductules and ducts during milking or suckling.

The application of ultrasound technology for the determination of milk compositions would offer several benefits that are not currently available with the use of on-farm equipment or standard laboratory testing. Ultrasound examination is a non-invasive, non-destructive method placing minimal stress on the animal [8]. Ultrasound imaging is easily accessible and portable ultrasound units can be used in developing countries or in rural areas that have limited access to laboratory services or are not willing to pay for shipping of multiple milk samples. Ultrasonographic equipment is affordable and examinations of the udder require little expertise. While specialized chemical laboratories that can determine basic milk composition (i.e., fat, protein, lactose, dry weight and ash content) often charge fair prices per sample, testing multiple samples can still be expensive. Additionally, the estimated cost of fatty acid profiling using gas chromatography can be in the tens to hundreds of dollars per sample. Commercial on-farm testing systems are an asset available to only large farms and producers; they require calibration and maintenance, and so a substantial investment must be committed. Lastly, determination of milk composition using ultrasound imaging could potentially be done on the day of examination, unlike standard laboratory testing, wherein the turnaround time is prolonged by shipping and other procedures that consume valuable time while the results are pending. Practically immediate determination of milk composition could be used to modify individual animals’ diets or management practices sooner than after laboratory testing [9].

There were two objectives of this study: (i) To determine quantitative echotextural characteristics of the mammary gland of lactating cows; and (ii) to assess the suitability of computerized image analysis of ultrasonographic images for determination of milk physicochemical properties in the cow, using echotextural variables as independent (predictor) variables. Our hypothesis was that quantitative ultrasonographic characteristics of the mammary gland parenchyma would correlate with milk chemical constituents and select physical parameters.

## 2. Materials and Methods

### 2.1. Animals, Location and Nutrition

All experimental procedures detailed below had been reviewed and accepted by the First Local Committee for Animal Use in Research (Cracow, Poland; #90/2012 and #22/2016). This study utilized thirty clinically healthy Holstein-Friesian cows (nine primiparous and 21 multiparous; mean body condition score of 2.8 [10] and body mass of 674 kg) housed in the experimental station of the Institute of Animal Genetics and Husbandry (IGiHZ) of the Polish Academy of Sciences situated in Jastrzębiec-Kosowo, Poland (latitude: 5249′59.880″ N and longitude: 153′0.000″ E). The Holstein-Friesian was chosen because it presently has the highest milk production of all dairy breeds worldwide. On the day of experiment, the cows were between day 58 and 222 of lactation with the average milk productivity of approximately 33 kg/day. Daily feed rations were balanced according to the Institut National de la Recherche Agronomique system [11]. In addition, rye grain of the “Bono” hybrid cultivar or the Dankowskie “Opal” cultivar (whole grain or shredded to 4.0 mm) was added to the total mixed ration (Table 1). 

### 2.2. Milk Sampling and Laboratory Analyses

The cows were milked mechanically twice daily, at 6 a.m. and 6 p.m. The milk yield of each cow was individually and electronically recorded at each milking. Pooled milk samples (from all the four udder quarters) from each of the 30 animals were then sent to the analytical laboratory. Collection, storage and transport of milk samples followed the PN-EN ISO 707 [12] directives. The samples (30–50 mL) were fixed with a preservative (Microtabs II, Bentley, Poland) and stored under refrigeration until analysis (physical properties). Milk samples for chemical composition analysis were collected to polypropylene containers with a Microtabs preservative and immediately transferred to the laboratory. The somatic cell count (SCC) was assessed with an IBCM device (Bentley Instruments, Chaska, MN, USA). Different parameters of milk samples (fat, total protein, casein, lactose, total solids, solids non-fat, citric acid, free fatty acids contents, urea level, acidity and freezing point) were evaluated using a MilkoScan FT2 device (Foss Analytical, Hilleroed, Denmark).

### 2.3. Ultrasonographic Scanning and Image Analysis

**Figure 1:**
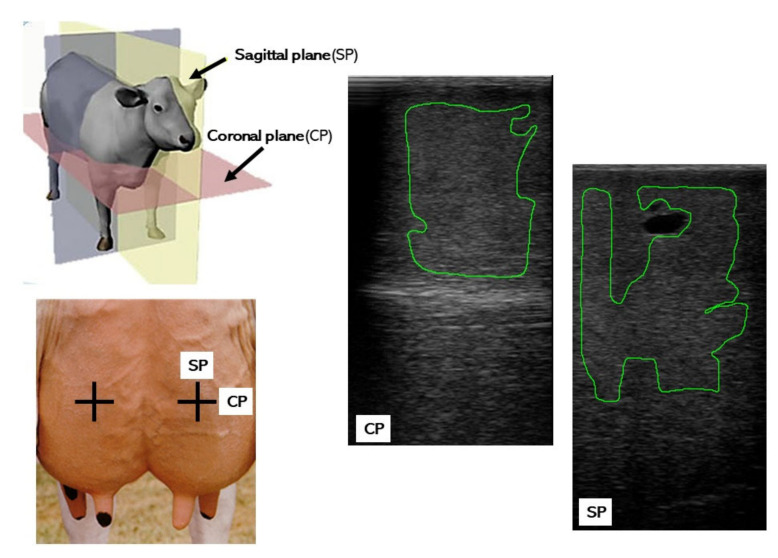
A general schematic of scanning planes and photographic reproductions of the mammary gland ultrasonograms from a Holstein-Friesian cow, obtained in a sagittal and coronal cross section, with outlines of polygonal areas of interest (green) excluding gland cisterns, blood vessels and artifacts, and used for computer-assisted analyses of mammary parenchyma echotexture.

Ultrasonographic scans of the cows’ udders were taken immediately before and after the milking, using a 5.0-MHz linear-array transducer connected to the Aloka ProSound 2 ultrasound scanner (Hitachi Aloka Medical Ltd., Tokyo, Japan). All cows were examined in a standing position, without sedation, by the same experienced operator. Whenever necessary, the hair over the udder was clipped to allow for the acquisition of good quality ultrasonographic images. Each of the four quarters of the udder was scanned in the sagittal and coronal planes, in the middle portion of the mammary gland (Figure 1). Hydrosoluble coupling gel was applied to the surface of the probe prior to ultrasonographic examination of each quarter. Throughout the study, all images were captured using the constant settings of the scanner for overall and near-far gain, contrast and focal points. A total of 960 ultrasonographic images were stored as DICOM (Digital Imaging and Communications in Medicine) files and then converted to grey-scale images (720 × 480 pixels resolution and 256 shades of grey) for echotextural analyses. The computerized image analysis was conducted in the Department of Biomedical Sciences at the University of Guelph, ON, Canada, using ImageProPlus^®^ analytical software (version 5.1 for Windows™; Media Cybernetics Inc., San Diego, CA, USA). In each ultrasonogram, a polygonal area of interest within the mammary gland parenchyma was selected avoiding reflection artifacts, visible blood vessels, lactiferous ducts, connective tissue and gland cisterns (Figure 1).Following area selection, the mean numerical pixel values (NPV) and pixel heterogeneity (pixel standard deviation-PSD) of each polygonal area were computed [13].

### 2.4. Statistical Analyses

The present data were analyzed using SigmaPlot™ computer software (version 11.0; Systat Software Inc., Richmond, CA, USA). A Student t-test was used to compare single time point observations (e.g., milk composition between the morning and evening sampling periods). The non-parametric Mann–Whitney Rank test was used for the data sets that failed the normality (Shapiro-Wilk) and/or equal variance tests. The echotextural variables (mean NPVs and PSDs) were compared using multivariate analysis of variance (ANOVA); a three-way ANOVA was performed to assess the main effects of time (morning vs. evening), scanning order (before or after milking) and scanning plane for each quarter (sagittal vs. coronal within right front vs. right back vs. left front vs. left back udder quarter). In addition, pixel intensities were normalized prior to statistical comparisons of udder echotextural variables by the ±3σ technique [14] to minimize the influence of scanning “noise” and image microartifacts. As there were no statistical differences among the raw and normalized data, the original values were used for ensuing statistical analyses. Pearson Product Moment test and simple linear regression were used for correlation analyses of echotextural parameters obtained in the coronal or sagittal plane with the physiochemical properties of milk during the morning and evening milking separately (due to significant differences in milk chemical composition). Correlations were determined for echotextural characteristics of the udder determined before milking. Statistical significance was declared at a *p*-value ≤ 0.05 in all analyses. All results are presented as mean ± standard error of the mean (SEM).

## 3. Results

### 3.1. Milk Yields and Composition

The amounts and physiochemical characteristics of the milk sampled both in the morning and evening are summarized in Table 2. There was a significant difference in the milk yield, total solids, solids non-fat, freezing point depression, acidity as well as crude fat and citric acid content; the values for those variables were higher in the evening, except for total milk yield and acidity, which were higher in the morning.

### 3.2. Factors that Impacted Mammary Gland Echotexture

There were significant main effects of the scanning order (before vs. after milking) as well as the scanning plane and udder quarter for both the NPVs and PSDs of the mammary gland parenchyma (Table 3).

Both the NPVs and PSDs were greater after than before milking (NPVs: 40.1 ± 0.3 compared with 38.7 ± 0.3; PSDs: 13.6 ± 0.07 compared with 13.2 ± 0.07; after compared with before milking, respectively; Table 4). Mean NPVs and PSDs for the left udder quarters (front and back) in the coronal plane were significantly greater than those for both right quarters scanned in the sagittal plane. The mean NPV and PSD values were 40.9 ± 0.6 and 13.7 ± 0.1 for the left front quarter coronal; 42.0 ± 0.7 and 14.0 ± 0.2 for the left back quarter coronal; 37.9 ± 0.7 and 13.1 ± 0.1 for right front quarter sagittal for 37.2 ± 0.6 and 12.9 ± 0.1 for right back quarter sagittal. Moreover, the left front quarter in the coronal plane had greater (*p* < 0.05) NPV and PSD values compared with the left front quarter in the sagittal plane (NPVs: 42.0 ± 0.7 compared with 38.7 ± 0.7; and PSDs: 14.0 ± 0.2 compared with 13.1 ± 0.1).

### 3.3. Correlational Results

There were four and six significant correlations among echotextural characteristics of the mammary gland parenchyma and physicochemical properties of the milk obtained in the morning and evening, respectively (Table 5). In the milk samples collected in the morning, significant correlations were recorded for protein and lactose content as well as freezing point depression, whereas during the evening milking the correlations were seen for protein, casein, lactose and solids non-fat. A vast majority of significant correlations were obtained for echotextural variables determined in a sagittal plane (3 correlations in the morning and 5 in the evening). Pixel heterogeneity of the mammary tissue was a primary ultrasonographic marker of milk physicochemical characteristics during the morning milking (3/4 correlations), whereas in the evening an equal number of significant correlations was seen for NPV and PSD. The strongest linear relationship was noted for PSD sagittal and milk protein content (chemical constituents) and for PSD coronal and freezing point depression (physical properties), both in the morning milking period.

## 4. Discussion

The main objective of this study was to examine the relationship between mammary gland echotexture and milk physicochemical characteristics in lactating cows. The effectiveness of ultrasound examinations paired with computerized image analysis for determination of tissue composition was confirmed by Ahmadi et al. [3] who demonstrated the existence of quantitative relationships between testicular tissue chemical composition and its echotextural attributes in rams. Testicular NPVs were negatively correlated with protein content while PSD was positively correlated with lipid content of the testicular parenchyma; computerized echotextural analyses could detect even small changes in tissue composition (i.e., ~1% of the crude fat or protein content; Ahmadi et al., [3]). It was, therefore, reasonable to suggest that the ultrasonographic technique coupled with computerized image analysis can potentially identify milk composition regardless of only the partial contribution of this excretory product compartment to the total udder content and its overall echotexture.

In the present study, morning milk yields were higher compared with the evening milking period while mean fat content was higher in the evening, which agrees with several previous studies [15,16,17,18]. In addition, milk samples obtained in the evening had higher total solids and solids non-fat percentages, citric acid content and acidity. Quist et al. [17] also observed that protein content was elevated during the evening milking of cows but we did not record such a difference in the Holstein cows of the present study; this could be partly due to the fact that in our experiment a 12-h milking interval was consistently used, whereas in the study by Quist et al. [17] the precise milking interval was not maintained or recorded. Regular monitoring of the variability in milk yields and physicochemical properties is important when making management decisions and in milk-recording programs of diary operations.

Mammary gland NPVs and PSDs were significantly greater after than before milking, which is in complete agreement with our earlier observations in low- and high-milk yielding sheep [13]. It is evident that the removal of the hypoechoic excretory product during complete milking alters the echotexture of the mammary parenchyma [19]. This is not surprising since 60 to 80% of milk in cows and 25 to 50% of milk in dairy sheep is stored in the mammary parenchyma [20,21]. Alternatively, Szencziová and Strapák [22] found that the difference in mammary gland echotexture before and after milking of cows was only minimal even though morphological changes associated with milk removal were significant (e.g., the elongation of the teat canal length after milking was estimated to 27% of the pre-milking length).

Interestingly, there was a significant difference in quantitative echotextural parameters between coronal and sagittal planes of the right and left udder quarters, respectively, and within the left front quarter in the cows of the present study. These results are puzzling and may suggest the existence of inherent differences in milk production, accumulation and/or composition among the four udder quarters in lactating cows. While Berglund et al. [23] found no differences in milk composition between the front and rear udder quarters of healthy cows, Sitkowska et al. [24] reported that it took 20–45 s longer to mechanically milk the rear quarters of the udder. Moreover, Andrée [25] found tremendous individual variations in milk yields and chemical composition between left and right quarters in cows.

To the best of the authors’ knowledge, this was a first attempt to correlate quantitative echotextural characteristics of the mammary parenchyma with select physicochemical properties of pooled milk samples. There is a great deal of evidence that ultrasound imaging combined with computerized image analysis can be used to predict histochemical characteristics of various internal organs and tissues. Some experimental and clinical studies have reported a link between echogenicity and tissue physicochemical properties (e.g., human dystrophic muscles [26], ram testes [3] and chicken pectoralis major muscle [7]). In 1998, Amin et al. [27] showed that image-processing analysis could be used to predict intramuscular fat content from ultrasound images obtained in live beef cattle. However, the accuracy of this method has been shown to decline when the percentage of intramuscular fat increases. One year later, a personal computer-based image analytical software, named USOFT, was developed to determine the percentage of intramuscular fat in live animals [27], but subsequently it has also been shown that USOFT software could accurately interpret the scanning results only in the range of 2–8% of intramuscular fat content [28]. It has recently been suggested that variations in milk yields and chemical composition between different genotypes of sheep may contribute to the differences in echotextural attributes of the mammary gland in lactating ewes [13]. In the present study, echotextural variables of the mammary parenchyma in cows, determined mainly in the sagittal plane, were significantly correlated with protein and lactose during both milking periods, with freezing point depression in the morning and casein and solids non-fat in the evening. Therefore, despite inconsistencies in significant correlations detected during the two milking periods, the resultant regression equations may provide the basis for developing a rapid, ultrasound-based technique of determining certain aspects of milk composition in commercial settings. As with the previous studies of skeletal muscles and gonadal tissue, the specific causative mechanisms of recorded correlations remain to be elucidated.

In animal production, ultrasonography has most frequently been used as a diagnostic tool. Transcutaneous examinations of the udder and teat are usually performed for detecting milk flow disturbances [29], measurements of the size of different udder compartments [30,31,32,33] and mastitis diagnosis [8]. Elevated somatic cell count (SSC) in the composite milk is an indicator of subclinical or clinical mastitis [34]. In goats, heterogeneity of the mammary parenchyma was significantly greater in animals with clinical mastitis compared with their counterparts with the subclinical disease and with clinically healthy controls [8]. However, we did not record quantitative relationships between parenchymal echotexture and SCC in the cows of the present study. It is feasible that sensitivity of computerized image analysis to detect changes in SCC is suboptimal for the range typical of subclinical mastitis; below the specific threshold, this technique may simply not be able to provide any predictions. Due to compartmentalized structure of the udder and dilution factor in the compose milk, one affected quarter may not be readily detected with SCC. Forsbäck et al. [34,35] found that even if SCC in composite milk is low (> 100,000 cells/mL), there is a risk of developing mastitis in individual quarters. According to Sharma et al. [36], SCC can be influenced by an array of factors including, but not limited to, the stage of lactation as well as animals’ age and parity. Significantly elevated SCC were also found in summer months [37]. Lastly, daily fluctuations in SCC of approximately 40% and occurring without any apparent reasons were reported in cows [36]. Future studies of the associations between mammary gland echotexture and SCC during clinical mastitis are warranted.

## 5. Conclusions

To recapitulate, our conclusions are as follows: (i) Milk in the udder contributes significantly to the echotexture on mammary gland parenchyma during the morning and evening milking periods of lactating cows (milked at regular 12 h intervals); (ii) there exist significant differences in quantitative echotextural characteristics among the four udder quarters and scanning planes (sagittal vs. coronal) due likely to intrinsic differences in milk yields and chemical composition within the udder; and (iii) mean NPVs and PSDs of the mammary gland parenchyma were related to the protein percentage, lactose content and freezing point depression determined in the milk samples collected in the morning, and to crude protein, casein, lactose and solids non-fat in the evening.

## Figures and Tables

**Table 1:** Ingredients, chemical composition and nutritive value of total mixed ration.

Item	Relative Content
*% of dry matter* Whole crop maize silage	35
Grass silage	22
Straw	5
High moisture corn grain silage	9
Concentrate mixture	28
*Concentrate mixture ingredients (%)* Rye grain of traditional/hybrid variety (ground to 4.0 mm) or rye grain of hybrid variety (unprocessed, whole grain)	42.0
Soybean meal	39.0
Canola cake	10.0
Fodder chalk	2.9
NaHCO_3_	2.8
Vitamix KW ^1^	2.6
Farmpak SC ^2^	0.7
*Chemical composition* Dry matter (DM, %)	48.2
*In dry matter (g/kg of DM)*	
Crude protein (CP)	147
aNDF ^3^	328
ADF ^4^	228
Starch	253
Crude ash	81
Crude fat	41
UFL ^5^ (in DM)	0.87
PDIN ^6^ (g/kg of DM)	95.9
PDIE ^7^ (g/kg of DM)	91.1

^1^ Mineral-vitamin additive (Polmass SA, Bydgoszcz, Poland); ^2^ Feed additive with active yeast *Saccharomyces cerevisiae* CNCM I-1077 (Levucell^®^ #174; SC; Lallemand Animal Nutrition, Milwaukee, WIS, USA); ^3^ Neutral detergent fiber determined with heat-stable amylase; ^4^ Acid detergent fiber; ^5^ Net energy for lactation; ^6^ PDIN-true protein absorbable in the small intestine when N is limiting in the rumen; and ^7^ PDIE-true protein absorbable in the small intestine when energy is limiting in the rumen.

**Table 2:** Summary of milk yields and laboratory testing of milk samples obtained from 30 Holstein-Friesian cows during the morning and evening milking periods. Ranges for each variable are given in parentheses.

Variable	Morning Milking	Evening Milking
Milk yield (l/cow) *	15.5 ± 0.7a (8.3–23.0)	10.4 ± 0.4b (4.8–14.9)
Somatic cell count* (×1000/mL)	365 ± 60 (35–997)	431 ± 65 (45–1110)
Fat (%)	3.3 ± 0.1a (1.7–4.6)	4.3 ± 0.1b (2.9–5.7)
Protein (%)	3.28 ± 0.05 (2.75–3.77)	3.38 ± 0.04 (2.81–3.78)
Casein (%)	2.29 ± 0.04 (1.87–2.65)	2.32 ± 0.03 (1.81–2.66)
Lactose (%)*	4.77 ± 0.03 (4.19–5.03)	4.77 ± 0.03 (4.25–5.03)
Total solids (%)	12.3 ± 0.1a (11.4–13.7)	13.3 ± 0.1b (12.2–15.5)
Solids non-fat (%)	8.96 ± 0.05a (8.35–9.47)	9.10 ± 0.05b (8.39–9.62)
Urea* (mg/l)	215.6 ± 13.1 (123–539)	215.1 ± 17.0 (104–622)
Citric acid (%)	0.13 ± 0.004a (0.09–0.18)	0.15 ± 0.005b (0.12–0.23)
Freezing point depression	547.2 ± 1.1a (537–563)	556.1 ± 1.3b (545–571)
Free fatty acids* (μg/mL)	0.67 ± 0.03 (0.29–0.99)	0.72 ± 0.04 (0.34–1.45)
Density (kg/m^3^)	1027.2 ± 0.3 (1025–1030)	1026.5 ± 0.2 (1024–1029)
pH	7.3 ± 0.1a (6.3–9.4)	6.7 ± 0.1b (5.8–8.2)

ab Within rows, mean values denoted by different letters vary significantly (*p* ≤ 0.05); * Data analyzed by the Mann–Whitney Rank Test.

**Table 3:** General Linear Model Analysis of Variance (ANOVA) table for the echotextural characteristics of mammary gland parenchyma (NPV: Numerical pixel values (0–255) and PSD: Pixel standard deviation or pixel heterogeneity) analyzed with three-way ANOVA.

Source of Variation/Echotextural Variables	NPV	PSD
Time of milking (morning vs. evening) (T)	0.08	0.58
Scanning order (before or after milking) (SO)	0.002	<0.001
Udder quarter + scanning plane (UQ + SP)	<0.001	<0.001
T × SO	0.35	0.06
T × UQ + SP	0.86	0.57
SO × UQ + SP	0.60	0.33
T × SO × UQ + SP	0.62	0.14

**Table 4:** A breakdown of the echotextural characteristics of the udder parenchyma (mean ± SEM) determined with computer-assisted analyses of mammary gland ultrasonograms from 30 Holstein-Friesian cows examined before and after complete mechanical milking in the morning and evening. NPV: Numerical pixel values (0–255); PSD: Pixel standard deviation or pixel heterogeneity. See text for details of statistical comparisons.

Time	Morning Milking		Evening Milking	
Scanning Order	Before	After	Before	After
Udder Quarter and Scanning Plane	Echotextural Variables			
	NPV			
Right front sagittal	38.1 ± 1.3	36.3 ± 1.3	38.8 ± 1.3	38.5 ± 1.6
Right front coronal	38.8 ± 1.1	39.4 ± 1.1	38.3 ± 1.3	40.3 ± 1.4
Right back sagittal	35.8 ± 1.0	39.0 ± 1.2	36.5 ± 1.0	37.3 ± 1.4
Right back coronal	38.3 ± 1.3	40.8 ± 1.2	40.2 ± 1.3	40.1 ± 1.4
Left front sagittal	38.0 ± 1.0	39.2 ± 1.5	37.2 ± 2.1	39.9 ± 1.5
Left front coronal	40.1 ± 1.1	40.0 ± 1.4	40.0 ± 1.3	43.2 ± 1.1
Left back sagittal	39.3 ± 1.4	39.1 ± 1.6	35.6 ± 1.1	42.2 ± 1.4
Left back coronal	39.5 ± 1.6	42.3 ± 1.7	41.3 ± 1.4	44.8 ± 1.1
	**PSD**			
Right front sagittal	12.9 ± 0.3	13.3 ± 0.3	13.5 ± 0.3	12.8 ± 0.3
Right front coronal	13.3 ± 0.2	13.8 ± 0.2	13.7 ± 0.3	13.4 ± 0.3
Right back sagittal	12.6 ± 0.2	13.6 ± 0.3	12.6 ± 0.3	12.8 ± 0.2
Right back coronal	13.4 ± 0.3	13.9 ± 0.3	13.2 ± 0.2	13.2 ± 0.3
Left front sagittal	12.7 ± 0.2	13.8 ± 0.3	13.0 ± 0.3	13.0 ± 0.3
Left front coronal	13.5 ± 0.2	13.8 ± 0.3	13.2 ± 0.2	14.2 ± 0.2
Left back sagittal	13.2 ± 0.3	13.3 ± 0.3	13.5 ± 0.3	13.6 ± 0.3
Left back coronal	13.7 ± 0.3	14.1 ± 0.3	13.6 ± 0.3	14.6 ± 0.3

**Table 5:** Correlations (Pearson Product Moment) among quantitative echotextural variables of the mammary gland parenchyma and physicochemical properties of milk samples collected during the morning and evening milking of 30 Holstein-Friesian cows. Correlations are for echotextural characteristics of the udder determined before milking. NPV: Numerical pixel value; PSD: Standard deviation of pixel values or pixel heterogeneity.

Input Variable (x)	Output Variable (y)	r	*p*-Value	Equation
Morning Milking				
NPV sagittal	Protein (%)	−0.39	0.03	y = 4.3 − 0.03x
PSD sagittal	Protein (%)	−0.42	0.02	y = 5.0 − 0.1x
PSD sagittal	Lactose (%)	0.36	0.05	y = 3.8 + 0.07x
PSD coronal	Freezing point depression	0.41	0.02	y = 510.0 + 2.7x
**Evening Milking**				
NPV sagittal	Casein (%)	−0.36	0.05	y = 2.9 − 0.02x
NPV sagittal	Solids non-fat (%)	−0.37	0.04	y = 10.0 − 0.02x
PSD sagittal	Protein (%)	−0.37	0.05	y = 4.3 − 0.07x
PSD sagittal	Casein (%)	−0.35	0.05	y = 0.6 + 1.2x
PSD sagittal	Solids non-fat (%)	−0.37	0.04	y = 10.1 − 0.08x
NPV coronal	Lactose (%)	−0.36	0.05	y = 5.3 − 0.01x
